# Hepatoprotective Activity of *Vitex trifolia* against Carbon Tetrachloride-induced Hepatic Damage

**DOI:** 10.4103/0250-474X.41466

**Published:** 2008

**Authors:** B. K. Manjunatha, S. M. Vidya

**Affiliations:** Department of Biotechnology, the Oxford College of Engineering, Bommanahalli, Bangalore-560 068, India; 1 Nitte College of Engineering, Nitte, India

**Keywords:** *Vitex trifolia*, aqueous and ethanol leaf extracts, CCl_4_, hepatoprotective activity

## Abstract

Aqueous and ethanol extracts of leaf of *Vitex trifolia* was investigated for hepatoprotective activity against carbon tetrachloride induced liver damage. To assess the hepatoprotective activity of the extracts, various biochemical parameters viz., total bilirubin, total protein, alanine transaminase, aspartate transaminase and alkaline phosphatase activities were determined. Results of the serum biochemical estimations revealed significant reduction in total bilirubin and serum marker enzymes and increase in total protein in the animals treated with ethanol and aqueous extracts. However significant rise in these serum enzymes and decrease in total protein level was noticed in CCl4 treated group indicating the hepatic damage. The hepatoprotective activity is also supported by histological studies of liver tissue. Histology of the liver tissue treated with ethanol and aqueous extracts showed normal hepatic architecture with few fatty lobules. Hence the present study revealed that *Vitex trifolia* could afford significant protection against CCl_4_ induced hepatocellular injury.

The plant *Vitex trifolia* L., (Verbenaceae) is commonly known as common chaste tree (English), *nochi* (Kannada) and *jalanirgundi* (Sanskrit). Leaves are commonly used as poultice for rheumatic pains, in inflammations, sprains and fever. Roots are used to treat febrifuge, painful inflammations, cough and fever. Flowers are used in treating fever and fruits in amenorrhoea^1^. This plant is known to possess various active constituents viz., essential oil^2^, halimane-type diterpenes, vitetrifolins^3^ and several pharmacological properties have been studied viz., antipyretic^4^, antibacterial^5^, against asthma and allergic diseases^6^. The plant is used by the local medical practitioners in treating acute jaundice. Literature survey revealed that, this plant has not been subjected to pharmacological screening for its hepatoprotective activity. This paper reports the hepatoprotective activity of *Vitex trifolia* against CCl_4_-induced hepatic damage.

Leaves of *Vitex trifolia* were collected from the Kuduremukha reserve forest of Chikkamagalur district, Karnataka State, during December 2003 and a voucher specimen (BKM-1012) is deposited in the Departmental Herbaria, Department of Botany, S. R. N. M. N. College of Applied Sciences, Shimoga, as authentic specimen for future reference.

Leaves were shade dried for a week, powdered mechanically (sieve no. 10/44) and stored in airtight containers. About 250 g of the powdered material was subjected to soxhlet extraction using 70% ethanol for 48 h. The solvent was distilled off at low temperature under reduced pressure using rotory flash evaporator. The yield was 24.5% w/w. Another 250 g of the powdered material was boiled in distilled water for 30 min, kept for 3 d with intermittent shaking, filtered and concentrated using rotory flash evaporator to get the aqueous extract. The yield was 17.5% w/w. Both the extracts were subjected to preliminary phytochemical tests^7^. Oral suspensions containing 20 mg/ml of ethanol extract and 30 mg/ml of aqueous extract were prepared in 1% w/v gum tragacanth.

Male Wistar rats weighing 150-200 g were procured from the National College of Pharmacy, Shimoga. The animals were housed in polypropylene cages and were maintained at 27±2°, relative humidity 60±5% and 12 h light/dark cycle, they were fed with commercial diet (Hindustan Lever Ltd., Bangalore) and water *ad libitum* during the experiment. The study was permitted by the Institutional Animal Ethical Committee with Reg. No. 144 /1999/ CPCSEA/ SMG/34.

Acute toxicity study was conducted for both the extracts by stair case method^8^. The LD_50_ of ethanol and aqueous leaf extracts were found to be 200 mg/kg p.o. and 300 mg/kg p.o. One tenth of this was selected as maximum dose for the evaluation of anti hepatotoxic activity^9^ i.e., 20 mg/kg p.o. and 30 mg/kg p.o.

The ethanol and aqueous extracts were selected for the evaluation of hepatoprotective activity against CCl_4_ induced hepatic toxicity. The animals were divided into five groups of six rats each. The animals in group I served as control and received the vehicle 1 ml/kg/day of 1% w/v gum tragacanth p.o., for 14 d. All the animals of group II to V received 0.1 ml/kg/day of CCl_4_ i. p. (E-Merck, Mumbai, India) for 14 d. Group III animals received the standard drug silymarin (Ranbaxy Lab, Dewas) in the dose of 100 mg/kg/day p.o., for 14 d. Ethanol and aqueous leaf extracts of *Vitex trifolia* were administered to the animals of group IV and V in the dose of 20 and 30 mg/kg/day p.o., respectively, for 14 d. The CCl_4,_ silymarin and the extracts were administered concomitantly to the respective groups of animals.

The animals of all the groups were sacrificed on 14^th^ day under light ether anaesthesia. The blood sample of each animal was collected separately by carotid bleeding into sterilized dry centrifuge tubes and allowed to coagulate for 30 min at 37°. The clear serum was separated at 3000 rpm for 10 min and was subjected to biochemical investigation viz., total bilirubin^10^, total protein^11^, serum alanine transaminase, aspartate transaminase^12^ and alkaline phosphatase^13^. Results of biochemical estimations were reported as mean±SE of six animals in each group. The data was subjected to one way ANOVA followed by Tukey's multiple comparison tests. P≤0.01 was considered as statistically significant.

The liver samples were excised from the experimental animals of each group and washed with the normal saline. Initially the materials were fixed in 10% buffered neutral formalin for 48 h and processed for paraffin embedding. The sections were taken at 5 μ thickness using microtome, processed in alcohol-xylene series and were stained with alum-haemotoxylin and eosin^14^. The sections were examined microscopically for the evaluation of histological changes.

Effect of ethanol and aqueous leaf extracts of *Vitex trifolia* on CCl_4_-induced liver damage in rats with reference to biochemical changes in serum is shown in the Table 1. At the end of 14 d treatment, blood samples of CCl_4_-treated animals showed significant increase in the levels of total bilirubin, alanine transaminase, aspartate transaminase and alkaline phosphatase compared to normal control groups but the total protein level decreased reflecting the liver injury caused by CCl_4_. Whereas blood samples from the animals treated with ethanol and aqueous leaf extracts of *Vitex trifolia* showed significant decrease in the levels of serum markers and significant increase in total protein to the near normal which are comparable to the values registered in the standard drug treated group of animals, indicating the protection of hepatic cells. Among the two extracts ethanol leaf extract showed significant protection against CCl_4_ induced hepatic damage.

**TABLE 1:** EFFECT OF ETHANOLIC AND AQUEOUS LEAF EXTRACTS OF *VITEX TRIFOLIA* ON CCL_4_-INDUCED HEPATOTOXICITY IN RATS

Group (N)	Total Bilirubin (mg/dl)	Total Protein (g%)	AST (IU/l)	ALT (IU/l)	ALP (IU/l)
Control	0.44±0.01	9.50±0.02	154.49±1.79	53.72±0.81	174.36±1.23
CCl_4_	2.50±0.04*	5.93±0.01*	2183.75±21.58*	1322.33±5.85*	442.10±2.07*
CCl_4_+silymarin	0.53±0.01**	8.69±0.06**	203.97±1.90**	74.11±1.41**	183.77±1.20**
CCl_4_+ethanol extract	0.63±0.01**	8.24±0.03**	217.38±1.08**	106.43±1.49**	202.47±1.72**
CCl_4_+aqueous extract	0.93±0.01**	8.05±0.03**	232.60±1.82**	124.17±0.85**	236.91±1.10**
ANOVA					
F	2195	1529	8238	3.82	5418
Df	4,25	4,25	4,25	4,25	4,25

N=6 animals in each group. Values are expressed as mean±SE.

* *P*≤0.01 indicates significant when compared to control.

** *P*≤0.01 indicates significant when compared to CCl_4_

Histological profile of control animal showed normal hepatocytes (fig. 1). The section of liver of the group II animals exhibited severe intense centrilobular necrosis, vacuolization and macro vesicular fatty changes (fig. 2). The liver sections of silymarin-treated animals showed normal hepatic architecture (fig. 3). The liver sections of the animals treated with aqueous extract exhibited moderate accumulation of fatty lobules (fig. 4). However significant liver protection was observed in the liver sections of ethanol extract treated animals as evident by the presence of normal hepatic cords, absence of necrosis with few fatty lobules (fig. 5).

**Fig. 1:**
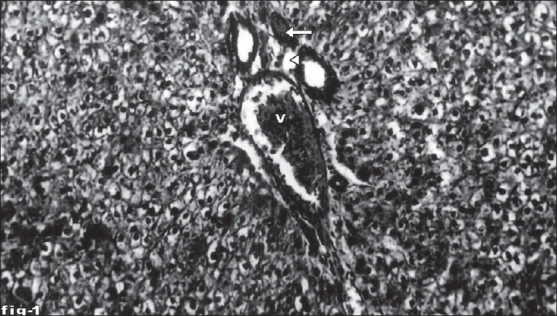
Liver tissue of control animal showing normal histology. Section of normal liver tissue with portal triad showing portal vein (V), portal artery (arrow) and hepatic duct (arrow head). Stain H and E, magnification 100X

**Fig. 2:**
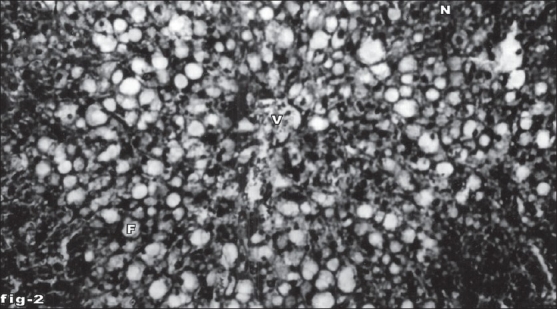
Liver tissue of animal treated with CCl_4_ showing necrosis. Section of the liver tissue of animal treated with CCl_4_ showing necrosis (N), fatty vacuole (F) and central vein (V). Stain H and E, magnification 100X.

**Fig. 3:**
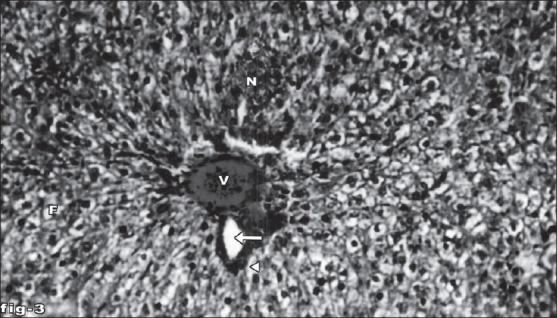
Liver tissue of silymarin-treated animals showing normal hepatocytes. Section of the liver tissue of silymarin-treated animals showing normal hepatocytes, portal vein (V), portal artery (arrow) and bile duct (arrow head). Stain H and E, magnification 100X.

**Fig. 4:**
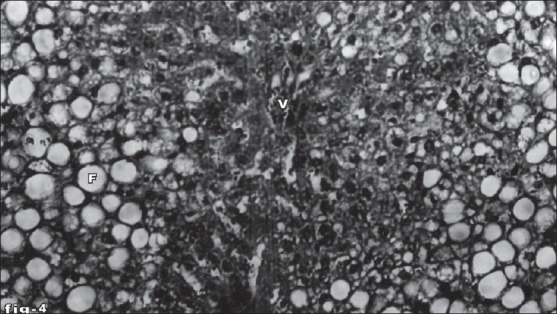
Liver tissue of aqueous leaf extract-treated animals showing normal arrangement of hepatocytes. Section of the liver tissue of aqueous extract of leaf-treated animals showing normal arrangement of hepatocytes around the portal vein (V), absence of necrosis and moderate accumulation of fatty vacuoles (F). Stain H and E, magnification 100X.

**Fig. 5:**
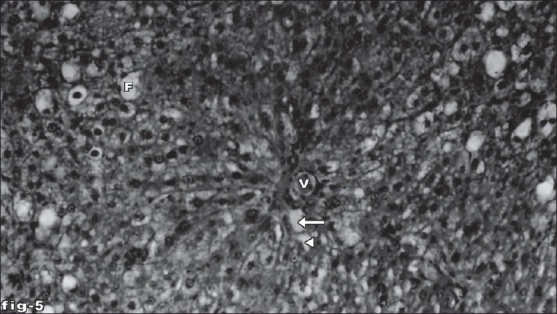
Liver tissue of ethanol extract-treated animals showing normal arrangement of hepatocytes. Section of the liver tissue of ethanol extract of the leaf-treated animals showing normal arrangement of hepatocytes around the portal vein (V), portal artery (arrow), bile duct (arrow head) and absence of necrosis and few fatty vacuoles (F). Stain H and E, magnification 100X.

CCl_4_-induced hepatic injury is the common model used for hepatoprotective drug screening. The extent of hepatic damage is assessed by the elevated level of biochemical parameters which is attributed to the generation of trichloromethyl free radical which in turn causes peroxidation of lipids of cellular membrane^15^. In the present investigation, preliminary phytochemical analysis of leaf extracts revealed the presence of flavonoids, tannins, saponins, glycosides, steroids and triterpenoids. Flavonoids^16^ and triterpenoids^17^ are well known for their antioxidant and hepatoprotective activities. In this study ethanol extract showed protective effect against toxicity induced by CCl_4_, which may be attributed to the individual or combined effect of antioxidant and hepatoprotective activity of phytoconstituents present in it. Based on the above results of the pharmacological screening, it can be concluded that the ethanol and aqueous leaf extracts of *Vitex trifolia* possesses significant hepatoprotective activity, which provides scientific evidence to the ethnomedicinal value of this rare plant genetic resource used by the tribal group of Western Ghats in treating jaundice.
